# Angiotensinogen Gene Missense Polymorphisms (rs699 and rs4762): The Association of End-Stage Renal Failure Risk with Type 2 Diabetes and Hypertension in Egyptians

**DOI:** 10.3390/genes12030339

**Published:** 2021-02-25

**Authors:** Islam M. El-Garawani, Eman M. Shaheen, Hesham R. El-Seedi, Shaden A. M. Khalifa, Gaber A. M. Mersal, Mahmoud M. Emara, Zeinab A. Kasemy

**Affiliations:** 1 Department of Zoology, Faculty of Science, Menoufia University, Menoufia 32511, Egypt; Eman.shaheen@science.menofia.edu.eg; 2 International Research Center for Food Nutrition and Safety, Jiangsu University, Zhenjiang 212013, China; 3 Biomedical Centre, Pharmacognosy Group, Department of Pharmaceutical Biosciences, Uppsala University, 751 23 Uppsala, Sweden; 4 Chemistry Department, Faculty of Science, Menoufia University, Menoufia 32511, Egypt; 5 Department of Molecular Biosciences, The Wenner-Gren Institute, Stockholm University, S-10691 Stockholm, Sweden; 6 Chemistry Department, College of Science, Taif University, Taif 21944, Saudi Arabia; gamersal@tu.edu.sa; 7 Department of Clinical Internal Medicine, Faculty of Medicine, Menoufia University, Menoufia 32511, Egypt; mahmoud.emara@med.menofia.edu.eg; 8 Department of Public Health and Community Medicine, Faculty of Medicine, Menoufia University, Menoufia 32511, Egypt; zeinab.kasemy@med.menofia.edu.eg

**Keywords:** angiotensinogen polymorphism, rs4762, rs699, hypertension, diabetic nephropathy, end-stage renal failure

## Abstract

Type 2 diabetes mellitus (T2DM) and hypertension are common chronic diseases mainly associated with the development and progression of end-stage renal disease (ESRD) leading to morbidity and mortality. Gene polymorphisms linked to the renin–angiotensin (*AGT*)–aldosterone system (RAAS) were broadly inspected in patients with diabetic nephropathy (DN) and hypertension. This study aimed to investigate the association of *AGT* gene polymorphisms (rs699 and rs4762) with ESRD in T2DM hypertensive Egyptian patients. Genotyping of rs699 and rs4762 was conducted using the tetra-primers amplification refractory mutation system (ARMS-PCR). The allelic distribution analysis was performed on 103 healthy control subjects, 97 non-ESRD patients, and 104 patients with ESRD. The allelic frequencies of *AGT* gene polymorphisms (rs4762 and rs699) in all study participants were assessed. For the non-ESRD group, the frequencies of the alleles of *AGT*-rs4762 (*χ*^2^ = 31.88, *p* < 0.001, OR = 5.17, CI 95%: 2.81–9.51) and *AGT*-rs699 (*χ*^2^ = 4.85, *p* = 0.027, OR = 1.56, CI 95%: 1.05–2.33) were significantly associated with the non-ESRD group. However, for the ESRD group, the T allele was significantly higher than that in the controls (*χ*^2^ = 24.97, *p* < 0.001, odds ratio (OR) = 4.35, CI 95%: 2.36–8.02). Moreover, *AGT* (rs699) genotypes showed no significant difference between the ESRD group and controls. In conclusion, *AGT* gene polymorphisms rs699 and rs4762 were associated with non-ESRD versus controls, without any significant risk observed in all patient groups. However, the *AGT* (rs4762) variant showed a significant risk in the ESRD group in comparison to controls in Egyptians.

## 1. Introduction

The most common etiology of chronic kidney disease (CKD) and the major cause of end-stage renal disease (ESRD) in adults is diabetic nephropathy (DN) in the Western world [1,2]. About 80% of patients with diabetic ESRD are affected by hypertension, which increases the rate of renal disease progression [3]. Therefore, in the case of various kidney diseases, interactions between multiple genetic and environmental factors are assumed to affect the progression of renal damage [4]. Renin–angiotensin–aldosterone system (RAAS) interruption is especially involved in ESRD progression and is one of the predisposing genetic factors [5]. RAAS is a central regulator of renal hemodynamics, volume homeostasis, and blood pressure [6]. The incidence of hypertension rises with diminishing renal functions [7]. This is due to both reduced sodium excretion and renin–angiotensin–aldosterone system (RAAS) activation. Therefore, it is possible to be linked with a genetic susceptibility to salt-dependent hypertension or over-activation of the RAAS. In patients suffering from ESRD, genes that control renal sodium reabsorption or RAAS genes may be highly significant [8]. 

Recently, the association of genes’ polymorphism with the progression of many diseases and drug responses has been investigated [9,10]. Many studies have reported mutations in the RAAS gene and polymorphisms causing host susceptibility to several diseases, including hypertension [11], type 2 diabetes [12], chronic kidney disease [13], and ESRD [14]. The *AGT* gene is considered as one of the candidate genes of the RAAS. It consists of four introns and five exons that are located on the long arm of chromosome 1 (1q42–43) [15]. As a part of the RAAS, *AGT* codes for 485 amino acids (AA) including the *AGT* chain (AA 34–485), a signal peptide (AA 1–33), and other peptides such as angiotensin I-II.

The possible effects of two missense single nucleotide polymorphisms (SNPs) of the *AGT* gene on DN were investigated in this study. The rs699 in exon 2 is a T-to-C exchange at codon 268 resulting in a functional replacement of methionine (M) to threonine (T) (M268 T). The rs699 was previously located at amino acid 235, and, therefore, it is also named M235T. In Japanese obese women, the polymorphism in *AGT* (rs699) has been reported to be associated with visceral obesity and hyperinsulinemia [16]. Another SNP on the *AGT* gene, rs4762, is a C-to-T substitution in exon 2 of the *AGT* gene at codon 207. As a result of this, the exchange of a functional threonine (T) to methionine (M), also known as T207M or T174M, occurs. The rs4762 may act as a predictor marker for the post-transplant diabetes mellitus development [17] in addition to essential and pregnancy hypertension [18,19]. Moreover, the risk for pregnancy-induced hypertension was also reported to be associated with rs4762 and rs699 haplotypes [18].

Several previous studies showed the relation between angiotensinogen gene polymorphism and ESRD development among either T2DM or hypertensive patients in populations other than Egyptians. In Egypt, the prevalence of T2DM is around 15.6% of all adults aged 20 to 79, and the prevalence of hypertension is approximately 26%. However, among ESRD patients, 15.5% had diabetes mellitus and 31.8% had hypertension [20,21]. As both diseases probably lead to ESRD, this study was designed to test whether the rs699 and rs4762 polymorphisms of the *AGT* gene are associated with the development of ESRD in T2DM and hypertensive Egyptian patients as a novel investigation linking both diseases with ESRD occurrence.

## 2. Subjects and Methods

### 2.1. Subjects

The present study on *AGT* gene polymorphisms was performed among 304 Egyptian subjects of both sexes and was divided into control subjects (*n* = 103), who were matched for age, sex and, socio-economic standards; patients’ group (*n* = 201), who were complaining of diabetes mellitus (T2DM) and hypertension and were recruited and distributed as non-ESRD group (*n* = 97) and ESRD group (*n* = 104). Written consent was obtained from the enrolled participants. Patients were enrolled from the Dialysis Unit, Faculty of Medicine, Menoufia University, while controls were recruited from the same population through invitations. All participants were received at a specially prepared laboratory center and all investigations were conducted for free. The study was approved by the Ethical Committee at the Faculty of Medicine, Menoufia University (No: 11/2020 INTM2). 

### 2.2. Methods

#### 2.2.1. Sample Collection

Samples of peripheral venous blood were collected in sterile EDTA-tubes (Kemiko Vacutainer, Egypt) for complete blood cell count (CBC) and genotyping assessments. Other aliquots of blood were collected in Kemiko Vacutainer sterile serum tubes (Egypt) for other laboratory investigations. Sera were separated by centrifugation and kept at −20 °C until use. All samples were labeled and numbered relevant to the numbers of other investigations.

#### 2.2.2. Laboratory Investigations

All patients had physical examinations and routine history taking. Complete blood cell count (CBC) using Sysmix KX–21 hematology analyzer (Sysmex Corporation, Osaka, Japan) and standard biochemical analyses, such as urea and creatinine (Biosystem, Barcelona, Spain) and electrolyte conditions (sodium, potassium, calcium, and phosphorus), were measured using an electrolyte analyzer (NS.BIOTEC EA-205, Alexandria, Egypt). Diabetes mellitus and hypertension were also monitored.

#### 2.2.3. Peripheral Blood Leucocytes’ Isolation

About 2 mL of anti-coagulated blood samples, within three hours of collection, were mixed (1:4 *v*/*v*) with erythrocyte lysing buffer (0.015 M NH_4_C1, 1 mM NaHCO_3_, 0.1 mM EDTA) for 20 min. at 30 °C. Then, centrifugation was performed for 5 min at 1500 rpm. Steps were repeated until the appearance of a white pellet of leucocytes [22]. The isolated pellets were kept at −80 °C until the DNA isolation step.

#### 2.2.4. Isolation of Total Genomic DNA 

The isolation of genomic DNA from the peripheral blood leukocytes was performed according to the extraction method of Aljanabi and Martinez [23]. The pellets of leukocytes were lysed using lysing buffer (50 mm NaCl, 1 mM Na_2_EDTA, 0.5% SDS, pH 8.3) for 2 h at 45 °C. Proteins and cellular contents were precipitated by 4 M NaCl, and nucleic acids were precipitated by cold isopropanol. The isolated palates were reconstituted in TE buffer (10 mM Tris, 1 mM EDTA and pH 8), and then stored at −20 °C until use.

#### 2.2.5. SNP Selection

Single nucleotide polymorphisms (SNPs) were selected according to PubMed published data (SNP database). In this study, SNPs were selected due to their significant association with the studied diseases [24,25,26,27].

#### 2.2.6. Genotype Assessment

To investigate the polymorphisms genotyping and allele analysis in the *AGT* gene (rs699 and rs4762), the tetra primers amplification-refractory mutation system (ARMS-PCR) was performed using a thermocycler Master cycler gradient (Eppendorf, Hamburg Germany). For rs699 and rs4762, DNA samples were initially denatured at 94 °C for 10 min, followed by 30 cycles of 94 °C of denaturation, 65 °C of annealing and 72 °C of extension for 1 min each. The primer sequences were illustrated in Table 1. 

For rs699, the product size for T and C alleles was 197 and 295 base pairs, respectively; however, the two outer primers (control) produced an amplicon with 448 bp. For rs4762, the products’ sizes were 198, 297, and 455 bp for C, T alleles and the control band, respectively. The limiting size of all fragments was selected within the range of 200–450 bp with an allelic band ratio of 1:5. The amplification reactions were performed in one reaction tube using the four primers simultaneously. The oligonucleotide primers were designed using primer design for tetra-primer ARMS-PCR (PRIMER1 online software, http://primer1.soton.ac.uk/primer1.html, accessed on 17 January 2021). The amplicons were resolved on 2% agarose gels (Sigma, St. Louis, MO, USA) and visualized on a UV trans-illuminator after staining with ethidium bromide [28].

#### 2.2.7. Statistical Analysis

Analyses were conducted using SPSS version 22.0 (SPSS Inc., Chicago, IL, USA). Patients’ demographic data are expressed as the mean ± SD. The significance of the association between the two groups was determined using Pearson’s chi-square (χ^2^) test. Fisher’s exact test was used when one of the expected cells was less than 5. ANOVA test was used to compare between more than 2 groups for parametric data. Test of homogeneity of variances was performed, and Tukey test post hoc analysis was used for assumed equal variance, while Dunnett T3 test was used for assumed unequal variance. Differences with a *p*-value of <0.05 are regarded as statistically significant. The 95% confidence interval (95% CI) and the odds ratio (OR) were calculated to evaluate the effects of variance. Hardy–Weinberg equilibrium (HWE) in patient groups and controls was tested. For additional analysis of the association between *AGT* gene polymorphisms (rs4762 and rs699) and the risk of disease, odds ratio was calculated in dominant, recessive, co-dominant 1, co-dominant 2, and over-dominant genetic models.

## 3. Results

### 3.1. Demographic and Clinical Characteristics

The characteristics of the patients with ESRD (*n* = 104), the patients with non-ESRD (*n* = 97), and the controls (*n* = 103) are shown in Table 1. The demographic and clinical data of this study revealed a non-significant difference between the studied groups regarding age or sex (*p* > 0.05). However, there was a significant difference between the controls and patient groups regarding all laboratory investigations (*p* < 0.01) except WBC (white blood cell) count. It was noticed that Hb, red blood cells (RBCs), platelets, Ca, Na, and albumin showed a significantly decreasing trend over the ESRD groups, w K, urea, and creatinine showed a significant increase. Albumin and platelets were significantly higher, while Na+ was significantly lower in patients of the non-ESRD group than those in the ESRD group (*p* < 0.001) (Table 2).

### 3.2. Genotyping of rs4762 and rs699

Hardy–Weinberg equilibrium (HWE) genotype frequencies of *AGT* gene (rs4762 and rs699) polymorphisms are demonstrated in Table 3. HWE was calculated for all studied SNPs. *AGT* (rs4762) genotype frequencies were matched with HWE among controls only, while they significantly differed from HWE among the two patient groups. *AGT* (rs699) genotype frequencies were matched with HWE among controls and the ESRD group, while they differed significantly from those expected by HWE among patients of the non-ESRD group. 

**Figure 1:**
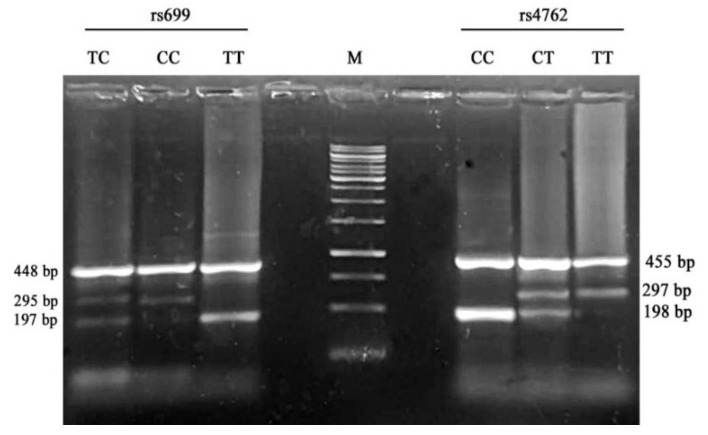
Representative digital photograph of ARMS-PCR products resolved on 2.0% agarose gel electrophoresis showing the *AGT* (rs699 and rs4762) genotyping; M, O’Gene Ruler™ 100 bp DNA ladders (Thermo Fisher Scientific, Austin, TX, USA).

The allelic frequency of rs4762 and rs699 was assessed using ARMS-PCR, and the products were resolved on agarose gel (Figure 1). Comparison between the allelic frequency of *AGT* genotypes in rs4762 and rs699 among patients’ groups and controls in different genetic models is demonstrated in Table 4 and Table 5. 

For the non-ESRD group (T2DM and Hypertension), the *AGT* gene polymorphisms (rs4762 and rs699) were significantly associated with the non-ESRD group in comparison to controls, where *AGT* (rs4762) genotypes were C/C (85.4% vs. 45.4%), T/C (14.6% vs. 51.5%), and T/T (0% vs. 3.1%) for controls and the non-ESRD group, respectively. Moreover, there was a significant difference between the two groups (*χ*^2^ = 33.17, *p* < 0.001, OR = 6.67, CI 95%: 3.37–13.17). The TC+TT combination served as a risk factor for the non-ESRD group and controls (*χ*^2^ = 35.75, *p* < 0.001, OR = 7.07, CI 95%: 3.59–13.92). For the T variant allele, there was a significant difference between the non-ESRD group and controls (*χ*^2^ = 31.88, *p* < 0.001, OR = 5.17, CI 95%: 2.81–9.51).

However, *AGT* (rs699) genotypes were T/T (22.3% vs. 6.2%), T/C (52.4% vs. 62.9%), and C/C (25.2% vs. 30.9%) for controls and the non-ESRD group, respectively. Additionally, a significant difference between the two groups (TC: *χ*^2^ = 9.74, *p* = 0.001, OR = 4.33, CI 95%: 1.64–11.43) and (TT: *χ*^2^ = 8.46, *p* = 0.0031, OR = 4.42, CI 95%: 1.56–12.52) was observed. The TC+CC combination served as a risk factor for the non-ESRD group and controls (*χ*^2^ = 10.50, *p* = 0.001, OR = 4.36, CI 95%: 1.69–11.25). For the C variant allele, there was a significant difference between the non-ESRD group and controls (*χ*^2^ = 4.85, *p* = 0.027, OR = 1.56, CI 95%: 1.05–2.33).

The *AGT* gene polymorphism (rs4762) was significantly associated with the ESRD group in comparison to controls, where the genotypes were C/C (85.4% vs. 49%) and T/C (14.6% vs. 51.0%) for controls and the ESRD group, respectively. Additionally, the allelic frequency showed a significant difference between the two groups (*χ*^2^ = 31.08, *p* < 0.001, OR = 6.10, CI 95%: 3.12–11.90). For the T variant allele, there was a significant difference between the ESRD group and controls (*χ*^2^ = 24.97, *p* < 0.001, OR = 4.35, CI 95%: 2.36–8.02). However, *AGT* (rs699) genotypes showed a non-significant difference between controls and the ESRD group. Comparison of the two patient groups revealed that *AGT* gene polymorphisms (rs4762 and rs699) did not show any significant difference (*p* > 0.05).

*AGT* polymorphic forms of rs4762 were significantly associated with the disease in the dominant (CC vs. CT+TT) (*p* < 0.001, OR = 6.55, CI 95%: 3.54–12.09), co-dominant 1 (CC^®^ vs. CT) (*p* < 0.001, OR = 6.36, CI 95%: 3.44–11.76), and over-dominant (CT^®^ vs. CC+TT) (*p* < 0.001, OR = 6.17, CI 95%: 3.34–11.39) forms. However, *AGT* (rs699) was significantly associated with the disease in the dominant (TT^®^ vs. TC + CC) (p = 0.047, OR = 1.85, CI 95%: 1.00–3.43), co–dominant 1 (TT^®^ vs. TC) (p = 0.046, OR = 1.91, CI 95%: 0.00–3.63), and over-dominant (TC^®^ vs. TT + CC) (*p* < 0.001, OR = 2.85, CI 95%: 1.64–4.96) forms.

## 4. Discussion

In this research, the associations between polymorphisms in the *AGT* gene (rs699 and rs4762), encoding for RAAS components, and the development of ESRD in diabetic and hypertensive cases were investigated.

Due to the increased RAAS activity, vasoconstriction and aldosterone release is initiated after angiotensin II induction, which subsequently expands the plasma volume, eventually leading to hypertension. Moreover, angiotensin II plays a vital role in hemodynamics, such as elevating the systemic and glomerular blood pressure and tissue growth enhancement, including mesangial hypertrophy and fibrosis development [29,30], which lead to the development of renal diseases. Another important point in this regard is the recent description of the intra-renal RAAS, which is believed to regulate the long-term blood pressure by affecting functions of tubular distant sodium-reabsorbing [31,32]. Recently, the relationship between RAAS gene polymorphisms (such as the rs669 and rs4762 gene polymorphisms) and ESRD, which is associated with circulating and cellular *AGT* concentrations, was also investigated regarding its involvement in ESRD etiology [33,34,35]. Angiotensin II can influence the metabolism of glucose via a cascade of pathways, including insulin signaling, adipogenesis, blood circulation, and oxidative stress [36,37]. 

In this study, significant elevation in urea and creatinine and alterations in electrolyte levels [38] associated with decreased Hb, RBCs, and platelets were expected to be observed in ESRD patients. This may be due to erythropoietin depletion affecting the erythropoiesis [39]. However, results of rs699 and rs4762 genotyping showed a significant association with the risk in the T2DM and hypertension groups in comparison to controls. However, *AGT* (rs4762) showed a significant risk in the ESRD group in comparison to controls (*p* < 0.05).

A previous study revealed that the rs699 variant genotype may be linked to the risk of diabetic nephropathy (DN) incidence in the Turkish population [24]. The polymorphic alleles of rs699 were associated with increased serum *AGT* levels in white patients and with the risk of increased blood pressure in both white and Asian patients [25] and were found to be linked to insulin sensitivity [40]. Furthermore, the association between a higher risk of DN and polymorphic allele T or TT genotype was investigated in two Indian populations [12,41], as well as Pakistani [42], Tunisian [43], Taiwanese [26], Japanese [44], Chinese [45] and Turkish populations [24]. However, the meta-analyses were unable to support the relationship between rs699 and DN [46,47,48]. Our results are in agreement with these findings, as we found an association between rs4762 and ESRD in T2DM and the hypertensive Egyptian population, while this was not the case for rs699. Moreover, our results are in agreement with the study conducted on Polish [49] and German populations regarding rs699 [50]. The association of rs4762 with DN has only been confirmed in the Taiwanese population [25], while in Slovenian T2DM subjects, it is not associated with DN [8]. Additionally, the polymorphic forms were found to be associated with the risk of T2DM in the Mewari population [27]. The current study is a cross-sectional study with a relatively small sample size. The results of our study cannot be generalized worldwide because only the Egyptian population was examined. Further studies including other genes associated with ESRD in T2DM hypertensive patients should be conducted with a larger sample size and mixed populations. The interaction between different risk factors, such as genetic, environmental, and racial among others, should also be considered.

## 5. Conclusions

*AGT* gene missense polymorphisms rs699 and rs699 were associated with non-ESRD in comparison to controls in our subset of the Egyptian population without any significant risk between patients’ groups (*p* < 0.05). However, *AGT* (rs4762) showed a significant risk only among patients in the ESRD group in comparison to controls. To the best of our knowledge, this is the first report on the genetics of ESRD with type 2 diabetes and hypertension among the Egyptian population. Further investigations and larger sample sizes will definitely provide further insight into this issue.

## Figures and Tables

**Table 1:** Primer sequences for rs699 and rs4762 detection using tetra primers amplification-refractory mutation system (ARMS-PCR).

**rs699**	**Forward**	**Reverse**
Outer	5′TGCGCACAAGGTCCTGTCTG3′	5′GTCACCAGGTATGTCCGCAGG3′
Inner	5′ATGGAAGACTGGCTGCTCCCTTAT3′ (T allele)	5′GCTGTCCACACTGGCTCACG3′ (C allele)
**rs4762**	**Forward**	**Reverse**
Outer	5′TTCCGTATATATGGCATGCACAGTGA3′	5′GAGCAGCCAGTCTTCCATCCTGT3′
Inner	5′GCCCAGCTGCTGCTGTCAAC3′ (C allele)	5′TGTGAACACGCCCACCAACA3′ (T allele)

**Table 2:** Demographic and laboratory data of the studied groups.

	Controls (*n* = 103)		>Non-ESRD (*n* = 97)		ESRD (*n* = 104)		*p-*Value
	Mean ± SD		Mean ± SD		Mean ± SD		
**Age (years)**	61.67 ± 6.30		62.69 ± 8.62		63.65 ± 4.21		0.099
**Sex (%):** Female Male	49 54	47.6 52.4	53 44	54.6 45.4	42 62	40.4 59.6	0.129
**MAP**	93.33 ± 1.32		96.57 ± 5.61 *		95.28 ± 8.36 *^a^		<0.001 ^#^
**HbA1c (%)**	4.68 ± 0.64		9.0 ± 1.71 *		8.49 ± 0.91 *^a^		<0.001 ^#^
**Hb (mg/dL)**	11.59 ± 1.82		11.35 ± 2.0		10.42 ± 1.86 *^a^		<0.001 ^#^
**RBCs × 10^6^**	3.92 ± 0.63		3.89 ± 0.71		3.59 ± 0.63 *^a^		<0.001 ^#^
**WBCs × 10^3^**	6.83 ± 2.31		6.98 ± 2.56		7.06 ± 2.38		0.788
**Platelets × 10^3^**	289.84 ± 73.04		247.83 ± 75.53 *		207.0 ± 64.71 *^a^		<0.001 ^#^
**PO_4_ (mg/mL)**	3.43 ± 0.37		3.33 ± 0.48		4.72 ± 1.26 *^a^		<0.001 ^#^
**Ca+ (mg/dL)**	8.54 ± 0.99		8.30 ± 0.98		7.09 ± 0.77 *^a^		<0.001 ^#^
**K+ (mEq/L)**	4.11 ± 0.27		4.0 ± 0.38		5.21 ± 0.74 *^a^		<0.001 ^#^
**Na+ (mmol/L)**	137.61 ± 1.72		137.64 ± 2.77		134.08 ± 4.40 *^a^		<0.001 ^#^
**Urea (mg/dL)**	28.60 ± 6.04		42.17 ± 12.99 *		132.89 ± 32.51 *^a^		<0.001 ^#^
**Creatinine (mg/dL)**	0.79 ± 0.11		1.06 ± 0.23		7.51 ± 1.581 *^a^		<0.001 ^#^
**Albumin**	4.38 ± 0.42		4.01 ± 0.33 *		3.71 ± 0.221 *^a^		<0.001 ^#^

#: Significance between all groups, *: significance between controls and non-end-stage renal disease (ESRD), ^a^: significance between non-ESRD and ESRD, chi-square test was used for qualitative data. One-way ANOVA test was used for quantitative data. Tukey HSD and Dunnett T3 tests were used for post hoc analysis. MAP: mean arterial pressure, HbA1c: glycated hemoglobin, RBCs: red blood cells, WBCs: white blood cells.

**Table 3:** Hardy–Weinberg equilibrium for *AGT* gene (rs4762 and rs699) genotypes among patient groups and controls.

Groups	*AGT* (rs4762)					*AGT* (rs699)				
	Genotype	Observed	Expected	χ^2^	*p* Value	Genotype	Observed	Expected	χ^2^	*p* Value
Control (*n* = 103)	CC^®^	88	88.5	0.635	0.425	CC	23	24.3	0.251	0.615
	CT	15	13.9			CT	54	51.5		
	TT	0	0.5			TT^®^	26	27.3		
Non-ESRD (*n* = 97)	CC^®^	44	49.1	6.31	0.011 *	CC	6	13.7	11.19	<0.001 *
	CT	50	39.8			CT	61	45.5		
	TT	3	8.1			TT^®^	30	37.7		
ESRD (*n* = 104)	CC^®^	51	57.8	12.15	<0.001 *	CC	21	25.0	2.47	0.115
	CT	53	39.5			CT	60	52.0		
	TT	0	6.8			TT^®^	23	27.0		

^®^ Reference, *: Significant, ESRD: end-stage renal disease.

**Table 4:** *AGT* gene (rs4762 and rs699) polymorphisms under the co-dominant and allelic models in patient groups and controls.

Variables	Control		Non-ESRD		χ^2^	*p*-Value	OR (CI 95%)
	No	%	No	%			
***AGT* (rs4762)**							
CC^®^	88	85.4	44	45.4	-	-	1.0
TC	15	14.6	50	51.5	33.17	<0.001 *	6.67 (3.37–13.17)
TT	0	0.0	3	3.1	5.74	0.040 *	-
CC	88	85.4	44	45.4			1.0
TC + TT	15	14.6	53	54.6	35.75	<0.001 *	7.07 (3.59–13.92)
**Alleles:**							
C^®^	191	92.7	138	71.1			1.0
T	15	7.3	56	28.9	31.88	<0.001 *	5.17 (2.81–9.51)
***AGT* (rs699)**							
TT^®^	23	22.3	6	6.2			1.0
TC	54	52.4	61	62.9	9.74	0.001 *	4.33 (1.64–11.43)
CC	26	25.2	30	30.9	8.46	0.003 *	4.42 (1.56–12.52)
TT	23	22.3	6	6.2			
TC + CC	80	77.7	91	93.8	10.50	0.001 *	4.36 (1.69–11.25)
**Alleles:**							
T^®^	100	48.5	73	37.6			1.0
C	106	51.5	121	63.4	4.85	0.027 *	1.56 (1.05–2.33)
	**Controls**		**ESRD**				
***AGT* (rs4762)**							
CC^®^	88	85.4	51	49.0			1.0
TC	15	14.6	53	51.0	31.08	<0.001 *	6.10 (3.12–11.90)
**Alleles:**							1.0
C^®^	191	92.7	155	74.5			
T	15	7.3	53	25.5	24.97	<0.001 *	4.35 (2.36–8.02)
***AGT* (rs699)**							
TT^®^	23	22.3	21	20.2			1.0
TC	54	52.4	60	57.7	0.31	0.58	1.22 (0.61–2.44)
CC	26	25.2	23	22.1	0.01	0.939	0.97 (0.43–2.19)
TT	23	22.3	21	20.2			1.0
TC + CC	80	77.7	83	80.7	0.14	0.706	1.14 (0.58–2.21)
**Alleles:**							
T^®^	100	48.5	102	49.0			1.0
C	106	51.5	106	51.0	0.01	0.919	0.98 (0.67–1.44)
	**Non-ESRD**		**ESRD**				
***AGT* (rs4762)**							
CC^®^	44	45.4	51	49.0			1.0
TC	50	51.5	53	51.0	0.10	0.753	0.91 (0.52–1.60)
TT	3	3.1	0	0.0	3.36	0.106	-
CC	44	45.4	51	49.0			1.0
TC + TT	53	54.6	53	51.0	0.27	0.601	0.86 (0.50–1.50)
**Alleles:**							
C^®^	138	71.1	155	74.5			1.0
T	56	28.9	53	25.5	0.58	0.445	0.84 (0.54–1.31)
***AGT* (rs699)**							
TT^®^	6	6.2	21	20.2			1.0
TC	61	62.9	60	57.7	7.08	0.007 *	0.28 (0.11–1.31)
CC	30	30.9	23	22.1	8.54	0.003 *	0.22 (0.08–0.63)
TT^®^	6	6.2	21	20.2			1.0
TC + CC	91	93.8	83	80.7	8.37	0.003 *	0.26 (0.10–0.68)
**Alleles:**							
T^®^	73	37.6	102	49.0			1.0
C	121	63.4	106	51.0	5.32	0.021 *	0.63 (0.42–0.93)

^®^ Reference, * significant value, OR: odd’s ratio, CI: confidence interval, ESRD: end-stage renal disease.

**Table 5:** Comparison between the two studied groups (all patients versus controls) according to *AGT* gene (rs4762 and rs699) polymorphism in different genetic models.

	OR (95% CI)	*p* Value
***AGT* (rs4762)**		
CC^®^ vs. CT + TT(Dominant)	6.55 (3.54–12.09)	<0.001 *
CC^®^ vs. CT(Co–dominant–1)	6.36 (3.44–11.76)	<0.001 *
CT^®^ vs. CC + TT(over dominant)	6.17 (3.34–11.39)	<0.001 *
***AGT* (rs699)**		
TT^®^ vs. TC + CC(Dominant)	1.85 (1.00–3.43)	0.047
TT + TC^®^ vs. CC (mutant)	1.06 (0.62–1.83)	0.832
TT^®^ vs. TC(Co–dominant–1)	1.91 (1.00–3.63)	0.046 *
TT^®^ vs. CC(Co–dominant–2)	1.74 (0.84–3.60)	0.135
TC^®^ vs. TT+CC (over dominant)	2.85 (1.64–4.96)	<0.001 *

^®^ Reference, ESRD: end-stage renal disease; OR: odds ratio; CI: confidence interval; *: significant.

## Data Availability

All data were included in the article.
